# High-Throughput Single-Molecule Microscopy with Adaptable Spatial Resolution Using Exchangeable Oligonucleotide Labels

**DOI:** 10.1021/acsnano.4c18502

**Published:** 2025-03-27

**Authors:** Klarinda de Zwaan, Ran Huo, Myron N.F. Hensgens, Hannah Lena Wienecke, Miyase Tekpınar, Hylkje Geertsema, Kristin Grußmayer

**Affiliations:** † Department of Bionanoscience, Kavli Institute of Nanoscience Delft, Delft University of Technology, 2629 HZ Delft, The Netherlands; ‡ Department of Imaging Physics, Delft University of Technology, 2628 CJ Delft, The Netherlands

**Keywords:** super-resolution imaging, single-molecule localization microscopy, fluorescence fluctuation imaging, DNA-PAINT, high-throughput microscopy, blinking kinetics

## Abstract

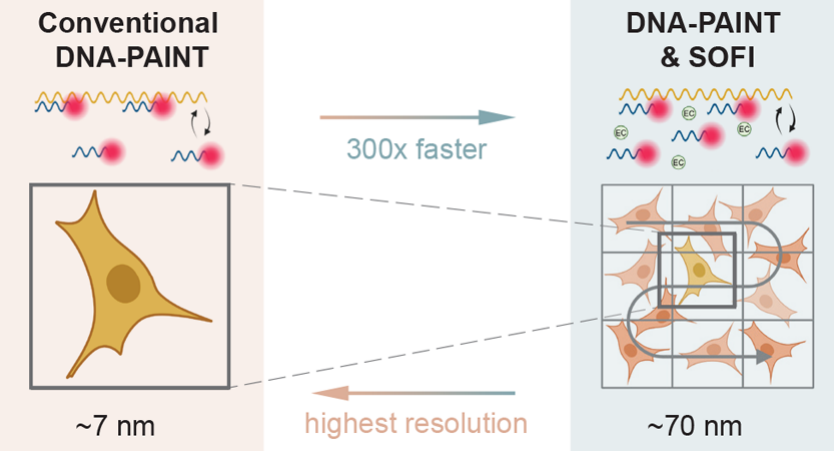

Super-resolution microscopy facilitates the visualization of cellular structures at a resolution approaching the molecular level. Especially, super-resolution techniques based on the localization of single molecules have relatively modest instrument requirements and are thus good candidates for adoption in bioimaging. However, their low-throughput nature hampers their applicability in biomolecular research and screening. Here, we propose a workflow for more efficient data collection, starting with the scanning of large areas using fast fluctuation-based imaging, followed by single-molecule localization microscopy of selected cells. To achieve this workflow, we exploit the versatility of DNA oligo hybridization kinetics with DNA-PAINT probes to tailor the fluorescent blinking toward high-throughput and high-resolution imaging. Additionally, we employ super-resolution optical fluctuation imaging (SOFI) to analyze statistical fluctuations in the DNA-PAINT binding kinetics, thereby tolerating much denser blinking and facilitating accelerated imaging speeds. Thus, we demonstrate 30–300-fold faster imaging of different cellular structures compared to conventional DNA-PAINT imaging, albeit at a lower resolution. Notably, by tuning the image medium and data processing though, we can flexibly switch between high-throughput SOFI (scanning an FOV of 0.65 mm × 0.52 mm within 4 min of total acquisition time) and super-resolution DNA-PAINT microscopy and thereby demonstrate that combining DNA-PAINT and SOFI enables one to adapt image resolution and acquisition time based on the imaging needs. We envision this approach to be especially powerful when combined with multiplexing and 3D imaging.

## Introduction

Fluorescence microscopy has elucidated important new insights into cellular processes over the past decades. The recent establishment of super-resolution microscopy methods has further pushed the boundaries of fluorescence microscopy to facilitate the visualization of cellular structures, such as nuclear pores and the neuronal cytoskeleton, at resolutions close to the molecular level.^1−3^ A particularly promising single molecule localization microscopy (SMLM) method is DNA-PAINT (point accumulation for imaging in nanoscale topography) since it can achieve the highest localization accuracy while still posing only modest hardware requirements. DNA-PAINT uses transient hybridization events between fluorophore-coupled single-stranded DNA oligonucleotides (imagers) and target-associated complementary DNA oligonucleotides (docking strands) that can be bound repetitively.^4^ The temporary immobilization of imagers yields a distinct fluorescent blink that can be computationally localized with an accuracy of a couple of nanometers. The oligo blinking kinetics are highly programmable by modulating DNA hybridization parameters through sequence design and buffer composition to obtain only a subset of the target-bound oligos to be hybridized to fluorescent imagers, thereby separating single blinking events in space and time.^5−11^ Localizations of tens of thousands of individual fluorescent blinks then render a super-resolved image. Compared to other SMLM techniques including dSTORM or PALM where photoswitching between on- and off-states are tuned by laser excitation, or chemical reagents, DNA-PAINT blinking events are solely determined by DNA oligo hybridization kinetics and thus uncoupled from fluorophore photophysics, allowing for the use of the brightest, photostable organic dyes.^11^ DNA-PAINT also facilitates the multiplexed imaging of different cellular structures in a single wavelength through the use of different imager–binder pairs sequentially, thereby avoiding chromatic aberration.^12^

Despite the many advantages of DNA-PAINT, this method is still sparsely deployed, arguably due to its low data throughput. The fluorescent signal of immobilized imagers needs to be segregated from diffusive background fluorescence by utilizing long exposure times of several hundreds of milliseconds, making DNA-PAINT the slowest super-resolution detection method. In addition, the cells cannot be visualized at low resolution prior to data acquisition, and because of the sparse fluorescent signal during a DNA-PAINT SMLM experiment, the underlying structure can be observed only after full data reconstruction. Consequently, significant time can be spent on the acquisition of data sets that do not result in informative images. This is a significant problem for expanding this promising visualization technique to advance our understanding of the nanoscopic organization of cellular molecules and is exacerbated in 3D imaging.

Here, we aim to improve the detection speed by combining DNA-PAINT with fluctuation-based imaging using super-resolution optical fluctuation imaging (SOFI) and to facilitate high-throughput super-resolution imaging. While both rely on the stochastic blinking of single fluorophores, SMLM and SOFI differ in the mechanisms exploiting information below the diffraction limit. SOFI uses higher-order statistical analysis of time series of blinking molecules (i.e., often a fluctuating signal from several overlapping fluorophores) to reconstruct super-resolution images and avoids the need to localize individual fluorophores. Correlations in the blinking signal allow us to perform spatiotemporal cumulant analysis with a moderate amount of frames to construct images with a super-resolution point-spread function raised to the power of the cumulant order n. SOFI is relatively insensitive to background signal, allowing for higher labeling densities, higher blinking on-time ratios, lower signal-to-noise, and reduced acquisition times than applicable for DNA-PAINT. Previous work by Glogger et al. showed that SOFI can be combined with exchangeable labels using standard DNA-PAINT to eliminate photobleaching effectively.^13^ Building on this, we utilize the programmability of DNA-PAINT kinetics and combine it with advanced SOFI processing to significantly speed up super-resolution imaging.

By tuning DNA-PAINT kinetics complemented with SOFI data processing, we establish super-resolution whole-cell imaging of microtubules and mitochondria with second-order SOFI in 5 s (500 frames) and up to fourth-order SOFI in 50 s (5000 frames), which is 30–300 times faster compared to SMLM data acquisition. Additionally, our approach allowed us to successfully achieve high-order SOFI up to the sixth order, with a resolution of 75 nm. Moreover, we also demonstrate that we can effectively switch between two super-resolution modalities, SOFI and SMLM. As a consequence, high-throughput imaging by SOFI can provide a quick sample overview at improved resolutions and deliver the necessary optical sectioning for, e.g., identification of rare phenotypes. Subsequent SMLM imaging of selected cells allows ultimate zoom-in at the highest resolution by leveraging the full resolving power of DNA-PAINT labels.

## Results and Discussion

### High-Resolution SOFI Using DNA-PAINT

**Figure 1:**
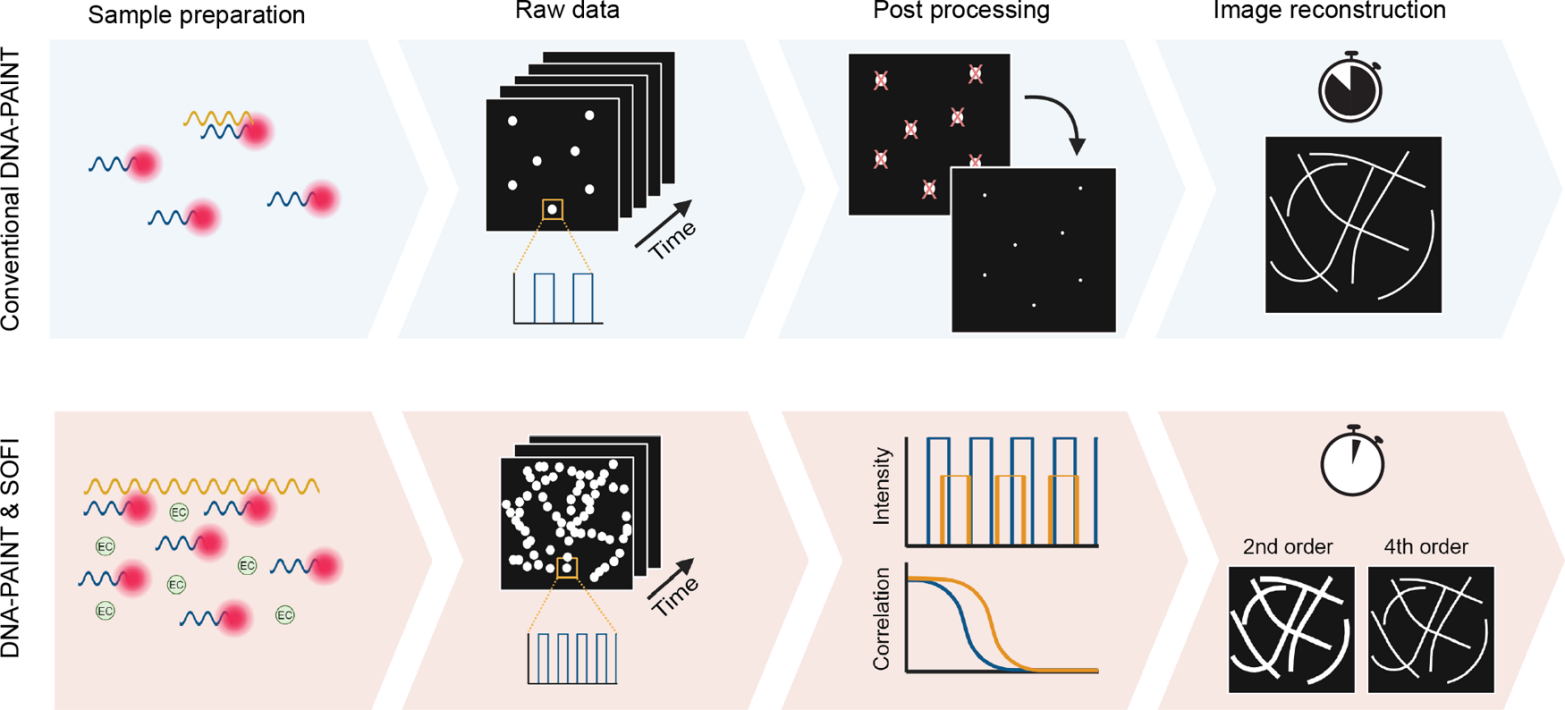
Overview of integration between DNA-PAINT and SOFI. The upper panel illustrates the conventional DNA-PAINT workflow, where a time series is recorded, individual emitters are localized with high accuracy, and a super-resolved image is reconstructed. The bottom panel demonstrates the integration between DNA-PAINT and SOFI. Here, optimized sample preparation is achieved through sequence design and buffer conditions to increase the frequency of fluorescent fluctuations, giving a higher emitter density per frame. SOFI calculations are then performed by using a pixel-by-pixel cross-cumulant algorithm combined with brightness linearization, producing high-order SOFI images. Fluorophores with different blinking kinetics (here: optimized and conventional DNA-PAINT sample preparation) result in different correlation patterns.

In this study, we explore the synergistic integration of DNA-PAINT and SOFI to accelerate super-resolution imaging. SOFI capitalizes on the fluctuations of fluorescent signals to achieve resolution beyond the diffraction limit (Supplementary Note 1).^14^ Measuring better blinking statistics will lead to a better SOFI signal, which is critical to exploit higher-order SOFI and thus higher spatial resolution. We focus on increasing the frequency of fluorescent fluctuations for SOFI analysis by adapting the binding kinetics of DNA-PAINT probes^15^ (Figure 1).

**Figure 2:**
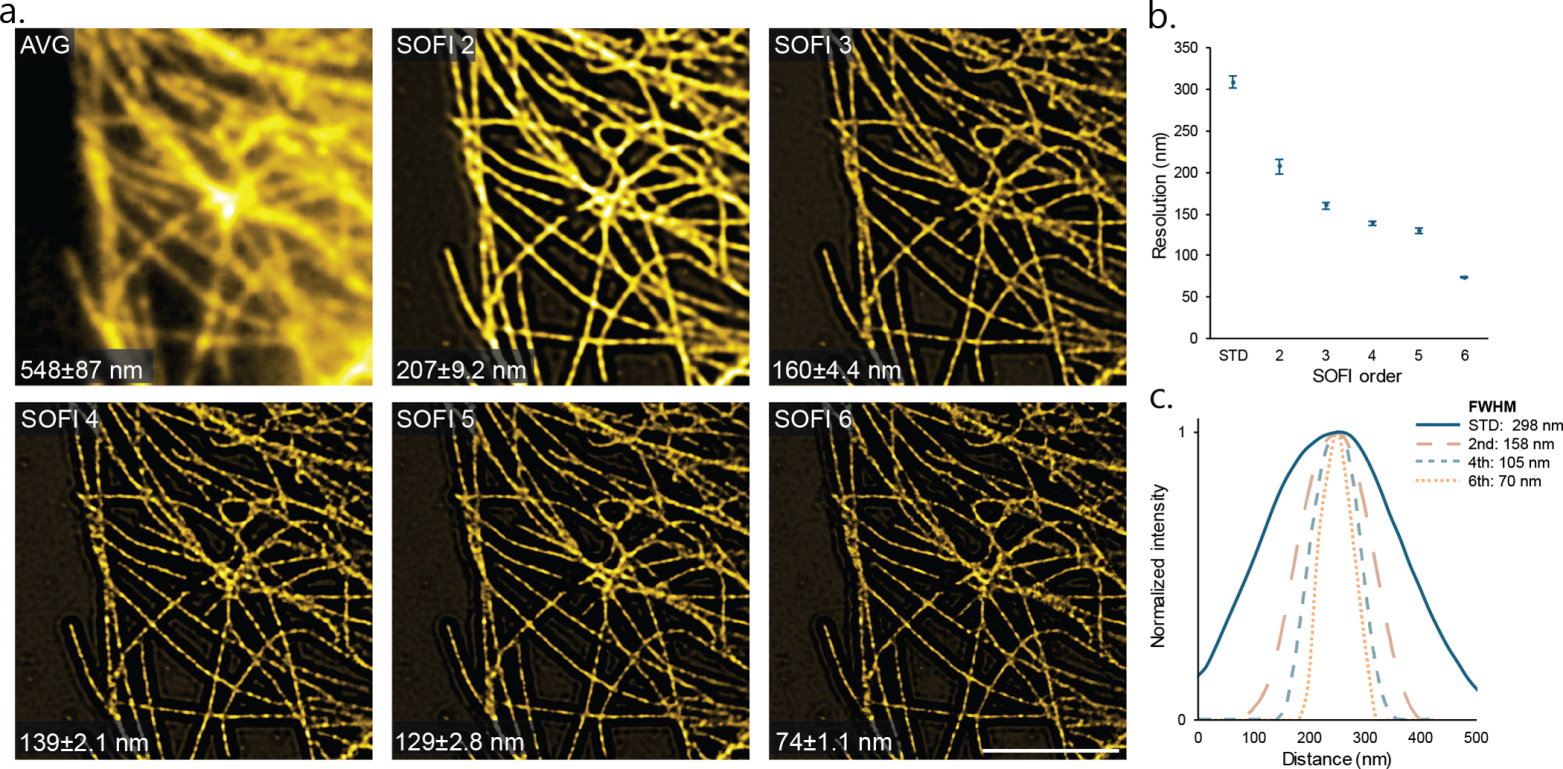
High-order DNA-PAINT SOFI reconstructions. COS-7 cells stained for microtubules with repeating docking sequence with the resolution increasing for increasing cumulant order. (a) Close-ups of diffraction-limited average projection of the image sequence, second-, third-, fourth-, fifth-, and sixth-order SOFI (scale bar 5 μm). (b) Resolution estimate by decorrelation analysis for three different cells for each order (average ± standard deviation).^19^ (c) Microtubule cross-section intensity profile for the standard deviation of the image sequence, second-, fourth-, and sixth-order SOFI and the corresponding fwhm measurements. See Table S1 for details about the imaging parameters.

In our experiments, we used speed-optimized DNA sequences with periodic sequence motifs (5xR1; see Table 1) that have been shown to provide a 5-fold increase in binding frequency^9^ compared to standard DNA-PAINT probes. In addition, we work with high imager strand concentrations in the nanomolar range to further raise the probability of hybridization (see Supplementary Note 2). Taken together, these experimental refinements allowed us to achieve up to the sixth-order SOFI of microtubules in fixed COS-7 cells shown in Figure 2. SOFI calculations were performed using a cross-cumulant-based algorithm with postprocessing including deconvolution and brightness linearization, which is essential for high cumulant orders^16−18^ (see Supplementary Note 1), thereby overcoming the limitations of Glogger et al. that obtained up to third-order reconstructions using conventional DNA-PAINT imagers.^13^

**Table 1:** Docking Site Sequences and Modifications for Nanobody Conjugation and the Corresponding Imager Strand Sequences

**Docking strand (5′–3′)**	
P3	azide - TTTCTTCATTA
5xR1	TCCTCCTCCTCCTCCTCCT - PEG4 - azide

**Imager strand (3′–5′)**	
P3	Atto655 - AGAAGTAATG
R1	AGGAGGA - Atto655

SOFI effectively suppresses background noise and improves optical sectioning; this is already apparent in the second-order reconstructions in Figure 2. In contrast, the average image shows prominent out-of-focus backgrounds, preventing the clear distinction between adjacent microtubule filaments. Each successive order *n* in SOFI contributes to resolving finer structural details, providing theoretically an up to *n*-fold resolution enhancement with subsequent deconvolution.^20^ To quantify the SOFI results and confirm the expected resolution increase with successive orders, the spatial resolution is estimated using image decorrelation analysis^19^ in Figure 2b and Supplementary Figure S5. The resolution enhancement is in good agreement with theoretical predictions (see Supplementary Figure S5). Specifically, for sixth-order SOFI, we achieve a remarkable resolution of approximately 75 nm. As a second metric for resolution, the intensity profile across the microtubule axis is quantified (Figure 2c). These results are consistent with decorrelation analysis, showing an increase in the resolution with higher orders. For the sixth order, the mean diameter of the microtubule (fwhm) is 70 nm. In addition, we show in Figure S3 mitochondrial structures that are also resolved up to sixth-order SOFI with the expected resolution enhancement, demonstrating the versatility of our approach.

Higher-order statistical analysis is challenged by the photophysical properties of the fluorophores used, limiting the usage of most fluorophores. First, the ideal fluorophore for SOFI should be photostable.^14,21^ Photobleaching, a correlated phenomenon, will affect the results and would need to be corrected for in the analysis. DNA-PAINT excels in this regard, as its blinking events are decoupled from the inherent fluorophore photophysics since imager strands are exchangeable and can be continuously replenished from a practically infinite buffer reservoir (see Supplementary Figure S6).^13^ Moreover, calculating higher-order cumulants requires well-sampled statistics and a homogeneous fluorescence blinking behavior^17^; both are the case for DNA-PAINT labels with fast fluctuations and uniform, programmed oligonucleotide binding–unbinding kinetics. Many fluorophores, however, exhibit inhomogeneous blinking during the measurement time, which limits their utility for analysis beyond second- or third-order SOFI and can lead to artifacts.^22,23^ In addition, DNA-PAINT probes enable the use of the brightest organic dyes. Altogether, DNA-PAINT labels tuned toward high fluctuations are particularly well-suited for high-order SOFI reconstructions due to their exceptional blinking behavior.

### Reducing the Acquisition Time

Next, our objective was to enhance the fluctuations to a level that allows us to increase the sampling rate and reduce the number of frames required, all while maintaining a high SOFI quality. To achieve this, we optimized our imaging buffer (Supporting Information, Note 2) by using even higher imager strand concentrations (with the periodic sequence motif docking strand) to decrease the off-time. At the same time, we added the small molecule ethylene carbonate (EC) to reduce the on-time by destabilizing the DNA duplex, leading to more pronounced intensity fluctuations. This approach still provided the blinking statistics required for achieving sixth-order SOFI (Supplementary Figures S3 and S4). But more importantly, these optimizations enabled us to measure at a higher frame rate of 100 Hz due to the greater frequency of binding and unbinding events.

**Figure 3:**
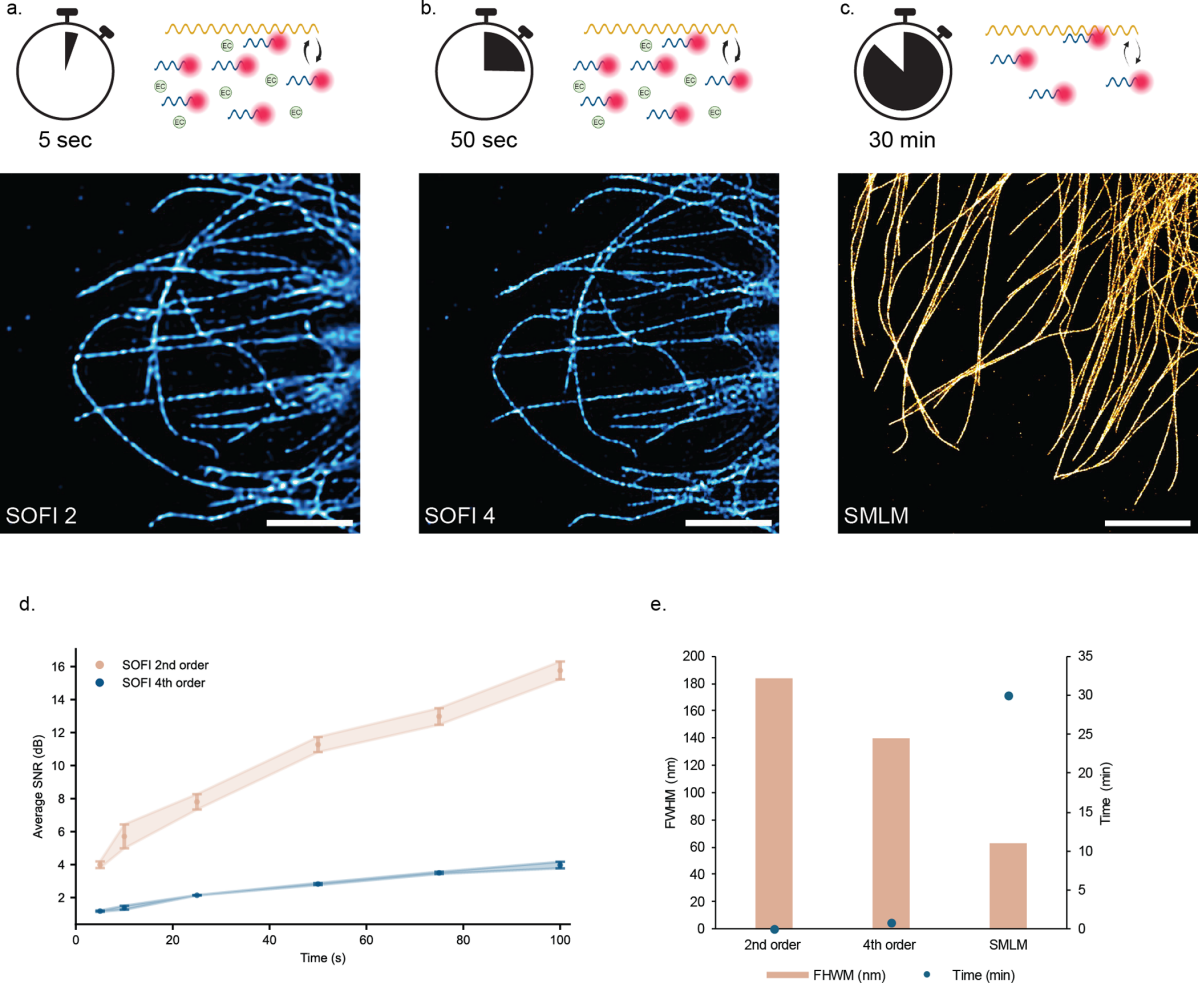
Minimum acquisition time for SMLM and SOFI DNA-PAINT. (a, b) SOFI DNA-PAINT reconstructions of fixed microtubules in COS-7 cells labeled with repeating docking sequence obtained with a minimal number of frames and with a frame rate of 100 Hz (scale bar 5 μm; see Supplementary Figure S7a,b for a qualitative assessment of minimal frame acquisition). (c) SMLM DNA-PAINT reconstructions obtained with a minimal number of frames and with a frame rate of 10 Hz (scale bar 5 μm; see Figure S7c for a qualitative assessment of minimal frame acquisition). (d) Jacknife SNR metric for second- and fourth-order SOFI as a function of acquisition time. (e) Average of five fwhm measurements of microtubule cross sections for each reconstruction correlated with the minimum acquisition time. See Supplementary Table S1 for details about the imaging parameters.

As a result, we reduced the acquisition time for second-order SOFI to only 5 s (or 500 frames) and for fourth-order SOFI to 50 s (or 5000 frames) (Figure 3a,b), which falls within previously reported ranges for other fluorophores.^17^ These achievements are validated through a qualitative assessment of structural continuity and the absence of artifacts for different acquisition times while the resolution is preserved (Supplementary Figure S7). Additionally, a pixel-wise SNR estimation based on a statistical approach known as jackknife resampling was performed^24^ to quantify the SNR of the SOFI images for different acquisition times (Figure 3d).

SMLM DNA-PAINT requires data acquisition at 10 Hz to ensure a sufficient signal-to-background ratio that can enable accurate localization. Localization of single emitters was executed, and we observed continuity in microtubule filaments after a time series of 30 min with a localization precision of 7 nm and an fwhm of 63 nm (Figure 3c,e). In comparison, our DNA-PAINT approach combined with SOFI analysis facilitated a 30-fold reduction in acquisition time for fourth-order SOFI (50 s) and a 300-fold reduction for second-order SOFI (5 s). The spatial resolution is estimated to be 208 nm for second-order SOFI and 151 nm for fourth-order SOFI, as determined using image decorrelation analysis,^19^ and 184 nm for second-order SOFI and 140 nm for fourth-order SOFI when evaluating the fwhm (Figure 3e). This highlights that, while SMLM achieves higher spatial super-resolution, it does so at the cost of increasing the acquisition time. Conversely, SOFI improves the acquisition speed (i.e., temporal resolution for dynamic samples) while still surpassing the diffraction limit, albeit with a reduced spatial resolution compared to SMLM. Thus, the SOFI optimized buffer allows for rapidly achieved super-resolution, marking a significant advancement in imaging speed and highlighting its potential for high-content super-resolution imaging applications.

### High-Throughput Super-Resolution Imaging for Screening Applications

**Figure 4:**
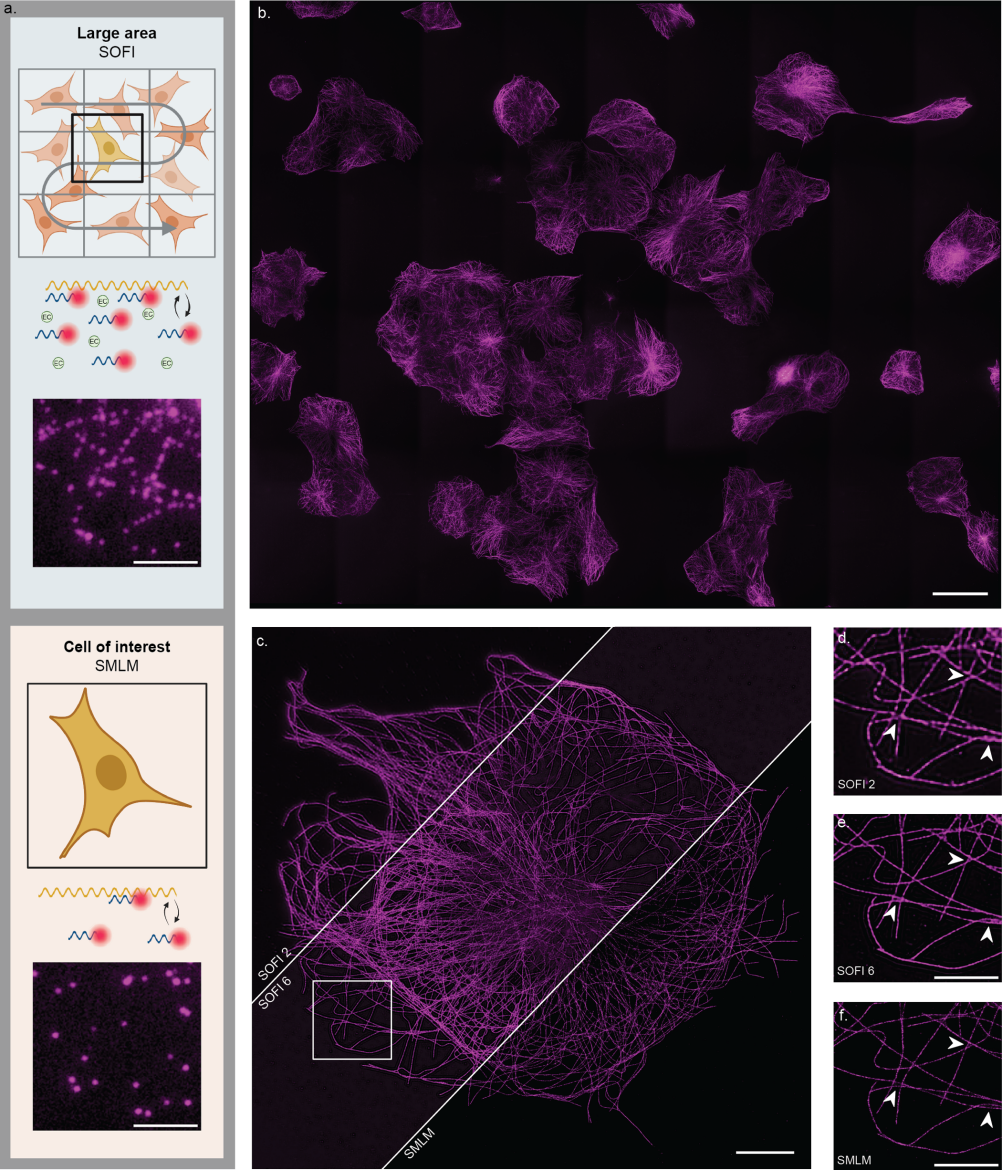
Proof of principle: High-throughput SOFI followed by SMLM. (a) Schematic of the proposed workflow. *Top:* A large area is imaged using a buffer optimized for fast SOFI. Example of raw data of COS-7 cells stained for microtubules for fast SOFI imaging (scale bar: 5 μm). *Bottom:* After washing away buffer components, a low concentration of imager strands is introduced, and a region of interest is imaged using SMLM. Example of raw data of COS-7 cells stained for microtubules for SMLM imaging (scale bar: 5 μm). (b) High-throughput second-order SOFI imaging of a 0.65 mm × 0.52 mm FOV containing about 40 cells (scale bar: 50 μm). (c) SOFI and SMLM reconstructions of the same FOV (scale bar: 10 μm). (d–f) Close-ups of the corresponding SOFI and SMLM reconstructions as indicated. Arrows indicate areas where improvement of resolution is visible (scale bar: 5 μm). See Supplementary Table S1 for details about the imaging parameters.

The drastic improvement in image acquisition time enables high-throughput super-resolution imaging by the SOFI directly, making it feasible to capture large areas efficiently (Figure 4a, top panel). To demonstrate, we acquire a 0.65 mm × 0.52 mm area by subsequently scanning partially overlapping FOVs in a 4 × 9 grid (Figure 4b). At each grid position, we cover an FOV of 84 μm by 150 μm and image for 500 frames at 10 ms exposure time. The total imaging time is thus 3 min plus an additional 1 min of stage movement and data saving time using an automated multiposition imaging protocol. Second-order SOFI reconstructs the microtubule network for the whole stitched FOV at a 2-fold resolution enhancement (Figure 4b).

To address the inherent trade-off between spatial and temporal resolution in traditional SMLM DNA-PAINT, we developed a workflow that integrates SOFI followed by SMLM. This approach leverages the fast data acquisition of SOFI to rapidly acquire super-resolution images, which are then used to guide subsequent imaging for SMLM. The sample remains unchanged, with only the buffer conditions modified to achieve the sparse blinking necessary for single-molecule localization (Figure 4a).

We imaged the same field of view with two different buffers (Figure 4c). We start with an SOFI optimized imaging buffer (i.e., high concentration of imager strands and EC) generating frames with a high density of emitters. To reconstruct a second-order SOFI image with 2-fold resolution enhancement (Figure 4d), we need 30 s of acquisition time per field of view, suitable for rapid screening applications as shown in Figure 4a. (Note: The longer acquisition time compared to the results shown in Figure 4a was due to our use of a reduced imager strand concentration. This adjustment was necessary to ensure thorough washing away of all imager strands within a reasonable time frame.) Based on the fast second-order SOFI image, we could then decide whether to continue imaging with the same buffer to achieve higher-order SOFI. By extending the acquisition to a total of 4 min, we obtained the blinking statistics necessary for sixth-order SOFI, which provides an expected 6-fold improvement in resolution (Figure 4e).

After these screening steps, if an area of interest or a specific event is identified that requires higher spatial resolution, we can transition to SMLM imaging. This involves washing away the initial imager strands and replacing the buffer with one that has a lower concentration of imager strands, which facilitates the sparse blinking, allowing localization of single emitters. We acquired SMLM data for a minimum of 25 min to reconstruct continuous microtubules with a localization precision of 8 nm (Figure 4f).

We used SQUIRREL to perform a comprehensive analysis of potential artifacts induced by SOFI processing in comparison to traditional SMLM DNA-PAINT processing (see Figure S8).^25^ The resolution-scaled Pearson (RSP) correlation coefficient and the root-mean-square error (RSE) between the reference and resolution-scaled images as a metric for structural discrepancies between the reference and super-resolution images indicate no major differences between SMLM and SOFI. However, we observed that the central part of the cell appears less resolved in SMLM than in SOFI, suggesting a potential advantage of SOFI in thick cellular regions.

This integrated workflow provides flexible, high-content imaging by combining the speed of SOFI for rapid screening with the high spatial precision of SMLM for more detailed analysis, making it highly adaptable to the specific demands of various experimental contexts. By optimizing the imaging process, we developed this approach for applications requiring both fast screening and high-resolution imaging. For instance, it will be particularly advantageous for screening large numbers of samples or cells to identify those suitable for downstream analysis or to recognize biologically rare events. Overall, the integration of SOFI and SMLM in our single-molecule-based super-resolution workflow significantly improves imaging efficiency without compromising resolution quality.

## Conclusions

In summary, our results showed the compatibility of exchangeable labels with two super-resolution techniques, DNA-PAINT and SOFI, and the advantage of a significant imaging speed increase when combining them. We also demonstrated spatial resolution tuning in high-throughput imaging with our method.

While both rely on the stochastic blinking of single fluorophores, DNA-PAINT and SOFI differ in the mechanism for extracting information below the diffraction limit. SOFI gains resolution enhancement from the statistical analysis of detectable fluorescence fluctuations. The quality of SOFI images depends on the effective contrast between on- and off-states, the SNR of acquired images, and the sampling of the blinking kinetics. Homogeneous blinking kinetics are beneficial for SOFI, and low photobleaching ensures that the spatial and temporal correlations analyzed in SOFI arise from stochastic fluorescence fluctuations.^17^

In this work, we used the exchangeable oligonucleotide-based probes and first sped up the blinking kinetics for SOFI using repeating sequences on the docking strand, which has been shown to increase the binding events' frequency.^9^ The highly correlative fluorescence fluctuations resulting from frequent binding and unbinding events at high imager concentrations are crucial for high-order SOFI analysis. We achieved the first successful sixth-order SOFI reconstruction of cellular structures with DNA-PAINT probes. This required the use of postprocessing algorithms including deconvolution and brightness linearization, resulting in the improvement of the sixth-order SOFI resolution up to 70 nm. Compared to a previous work utilizing exchangeable nucleotide-based probes for SOFI,^13^ this increased the resolution enhancement by approximately 3-fold and is in line with the best resolution reported in imaging continuous cellular structures for SOFI, which is 50–60 nm at sixth order.^17^

Similar to other SMLM techniques, conventional DNA-PAINT suffers from a long acquisition time. Advances in speeding up DNA-PAINT have been focusing on accelerating the blinking kinetics, i.e., shortening both the on- and off-times of blinking fluorophores. However, shorter on- and off-times translate to more fluorophores present in each frame, which poses challenges for SMLM due to the overlapping PSFs in a dense frame that lead to image artifacts. In addition, the localization precision suffers from shorter acquisition times due to a reduced signal-to-background ratio. SOFI eliminates the requirement of sparse distinguishable fluorophores, thereby opening up more blinking kinetics space for imaging speed-up. We combined several strategies to increase the blinking frequency. Next to using repeating sequences on the docking strand, we simply increased the imager strand concentration to increase the binding event frequency and to decrease the off-time. We also added EC to the imaging buffer,^26^ which destabilizes the DNA duplex in order to increase the dissociation rate and to decrease the on-time. This resulted in a second-order SOFI image of the microtubule network in cells within only 5 s, or 500 frames at 100 Hz. Our data acquisition is 25-fold faster compared to that in Glogger et al.,^13^ where the total acquisition time amounts to 125 s using the standard P1 and P4 sequences. Obtaining more frames for as long as 50 s facilitated the fourth-order SOFI reconstructions in our measurements, 30-fold faster than a typical SMLM acquisition.

Compared to other methods to accelerate DNA-PAINT, e.g., argo-PAINT^8^ and FLASH-PAINT,^27^ we avoided adding additional protein or nucleic acids or greatly extending the imager strand length. Our approach could also be combined with other factors in the buffer affecting the nucleic acid binding kinetics, such as salt concentration and temperature.^28^ The limitation of our method for more acceleration is mainly the high background signals at higher imager strand concentration that eventually compromise the SNR, even though SOFI intrinsically suppresses the noncorrelative background noise. We used TIRF or HILO illumination for our images to provide extra optical sectioning that facilitated higher-order SOFI. The upper limit of acceleration supported by increased blinking kinetics depends on the structures of interest and the docking strand labeling efficiency. The recent fluorogenic and self-quenched imager strands^7,29^ can further help to reduce background and expand applications in thick samples. 3D SOFI where different *z*-positions are imaged at the same time, for instance, through multiplane splitting,^20^ can further increase the throughput of the approach.

The optimization of spatial resolution and the acquisition time of DNA-PAINT-SOFI not only increase the imaging speed at high resolution and high throughput but also can function as a useful tool for fast high-content screening of samples at a moderate resolution enhancement. The drastic reduction in acquisition time allowed for a 4 min imaging with 2-fold resolution enhancement, scanning through a total FOV of 0.65 mm × 0.52 mm. We demonstrated that we can conveniently switch from SOFI conditions to SMLM with localization precision of a few nanometers, simply by modifying the buffer composition, i.e., by lowering the imager strand concentration. The resolution improvement between fluctuation-based (about 70 nm) and localization imaging (about 7 nm) in our workflow is akin to switching between confocal and STED imaging, which is routinely performed to facilitate data acquisition.

Our second-order SOFI acquisition time for a single position is of a similar scale as structured illumination imaging with DNA-PAINT labels^30,31^ that enables a maximum 2-fold resolution increase. Importantly, our SOFI to SMLM workflow can be carried out using a microscope with simple hardware, facilitating the straightforward adoption of our proposed approach. We envision screening a large number of cells with fast SOFI and using for example, machine learning algorithms to interrogate the optically sectioned and background-reduced images to identify rare phenotypes for subsequent interrogation by DNA-PAINT. Since DNA-PAINT relies on stochastically blinking single molecules, identification of full protein structures or networks, and thereby rare events, is generally hampered by time-intensive image acquisition. In fact, the continuous adjustment capability of blinking kinetics with exchangeable oligonucleotide-based probes facilitates the tuning of temporal and spatial resolutions to visualize protein structures and networks from a few nanometers with SMLM to dozens with SOFI.

Finally, this approach, which involves controlling the blinking dynamics, is not limited to fixed cells alone. Novel PAINT-alike probes compatible with live cells, such as self-labeling protein tags labeled with reversible fluorescent probes,^32^ offer a promising outlook for high-content live-cell super-resolution imaging. We envisioned our method contributing toward the goal of fast 3D multitarget super-resolution imaging.

## Methods and Experimental Section

### Nanobody Production

Bacterial expression plasmids pTP1122 and pTP955 and pDG02583 were a gift from Dirk Görlich (Addgene plasmid #104159; http://n2t.net/addgene:104159; RRID:Addgene_104159, Addgene plasmid #104164; http://n2t.net/addgene:104164; RRID:Addgene_104164, Addgene plasmid #104129; http://n2t.net/addgene:104129; RRID:Addgene_104129, respectively).^33^

The antimouse and antirabbit nanobodies with protease-cleavable affinity tags and engineered cysteines, and *bd*NEDP1 protease fused to His14-MBP-*bd*SUMO, were expressed in *E. coli* BL21(DE3).^33^ 2 L of Luria–Bertani broth (LB broth) was inoculated with 20 mL of overnight culture. *E. coli* were grown to an OD600 between 0.4 and 0.7 before protein expression was induced by 0.5 mM isopropyl B-D-1-thiogalactopyranoside (IPTG). 4 h after induction, cells were pelleted by centrifugation and resuspended into lysis buffer (50 mM Tris/HCL; pH 8.0, 1 M NaCl, 5 mM beta-mercaptoethanol, 50 mM imidazole) and 1 mM PMSF was added. Cells were lysed by sonication, and the lysate was cleared by ultracentrifugation for 30 min at 4 °C (Ti45 rotor, 37,000 rpm, Beckman Coulter). The proteins were purified by affinity chromatography using an ÄKTA Start (GE Healthcare ÄKTA Start). The lysate was passed through a 5 mL pre-equilibrated HisTrap HP column (Cytiva) and was washed with lysis buffer. Gradient elution was performed over 10 column volumes (CVs) with a filter sterilized elution buffer (50 mM Tris/HCl; pH = 8.0, 150 mM NaCl, 5 mM beta-mercaptoethanol, and 500 mM imidazole). The fractions containing the nanobody were pooled, and a buffer exchange to maleimide labeling buffer (MLB; 100 mM potassium phosphate buffer; pH = 7.5, 150 mM NaCl, 250 mM sucrose) using SnakeSkin dialysis tubing was performed. For the *bd*NEDP1 protease, the eluate was rebuffered to protease buffer (50 mM Tris/HCl; pH = 7.5, 300 mM NaCl, 250 mM sucrose). The protein concentration after buffer exchange was determined by using a Nanodrop1000 spectrophotometer (Thermo Fisher Scientific). The nanobodies and protease were aliquoted, frozen in liquid nitrogen, and stored at −80 °C until further use.

1 mM His-tag containing nanobodies was cleaved by 0.6 μM bdNEDP1 protease in a thermoshaker at 20 °C and 300 rpm for 24–96 h. Cleaved His-tags, His-tag containing proteases, and uncleaved nanobodies were purified out of the solution by reverse affinity chromatography using an ÄKTA Start (GE Healthcare ÄKTA Start). The cleaved mixture was subjected to purification using a pre-equilibrated 1 mL HisTrap HP column (Cytiva). After loading the sample, the column was washed with MLB to separate and collect the unbound protein (i.e., cleaved nanobodies). Subsequently, gradient elution was conducted over five CVs using MLB supplemented with 500 mM imidazole. During elution, the cleaved tags and proteases were collected. The purity of the nanobodies was assessed by using SDS-PAGE, and the protein concentrations were measured using a Nanodrop1000 spectrophotometer (Thermo Fisher Scientific). Subsequently, the cleaved and purified nanobodies were aliquoted, frozen in liquid nitrogen, and stored at −80 °C until further use.

### Sample Preparation

#### Site-Specific Labeling of Nanobodies

A site-specific labeling protocol of the nanobodies with an azide functionalized DNA oligonucleotide was developed based on the literature and containing a two-step reaction.^26,34^ First, a DBCO-maleimide linker is conjugated to the nanobodies with engineered cysteines. Second, 5′-azide functional oligonucleotide docking strands are conjugated.

Purified and cleaved nanobodies with engineered cysteines were freshly reduced with a 30-fold molar excess of 15 mM TCEP (Carl Roth) for 30 min on ice. For a standard reaction, 40 μM reduced nanobody was mixed with 2 mM DBCO-maleimide (Jena Bioscience) and incubated for 4 h at 4 °C. Unbound reaction partners were removed in two buffer exchange steps with phosphate-buffered saline (PBS; Gibco, ThermoFisher) using a Zeba spin desalting column (10,000 MWCO). The protein concentration and the degree of labeling (DOL) were determined by absorbance at 280 nm for the nanobodies and at 309 nm for DBCO using a Nanodrop1000 spectrophotometer (Thermo Fisher Scientific).

Docking strand-oligonucleotides (see Table 1), modified with either a 3′ or a 5′ azide moiety, were synthesized by Biomers.net (Germany) and dissolved in PBS to a concentration of 5 mM. For a standard reaction, 10 μM nanobody was incubated with 300 μM azide-docking strand for 30 min at 20 °C at 300 rpm. Unconjugated docking strands were removed similar to the DBCO conjugation using a Zeba spin desalting column (10,000 MWCO), and protein concentration was determined by measuring the absorbance at 280 nm. The conjugated nanobodies were stored either at 4 °C in PBS or at −20 °C in 50% glycerol.

#### Cell Culture

COS-7 cells (DSMZ GmbH) were cultured in Dulbecco’s modified Eagle's medium with high glucose (ThermoFisher) supplemented by 10% fetal bovine serum (Gibco, ThermoFisher), 1% sodium pyruvate (Gibco, ThermoFisher), 1% l-glutamine (Gibco, ThermoFisher), and 1% Penicillin-Streptomycin (Gibco, ThermoFisher). Cells were cultured in a 10 cm culture dish and incubated at 37 °C and 5% CO_2_. Cells were passed twice a week at 90% confluence, by washing with PBS, incubating with Trypsin/EDTA (Gibco, ThermoFisher) for 3–5 min at 37 °C, and diluting the cells in a fresh medium (1:10) on a new plate.

COS-7 cells were seeded either on 24 mm high-precision cover glasses (Carl Roth) in a six-well plate or on μ-Slide 8 Well high Glass Bottom (Ibidi). The cover glasses were first plasma-cleaned by exposure to O_2_-plasma for 2 min, making the surface hydrophilic, and allowing better adhesion of cells for microscopy experiments. Cells at 90% confluence were appropriately diluted at a 1:10 ratio and seeded onto the substrates. Following seeding, the cells were incubated at 37 °C and 5% CO_2_ overnight followed by fixation procedures.

#### Immunostaining

COS-7 cells were fixed when a moderate confluence of single cells was reached. Generally, this means that the samples were fixed about 24 h after seeding. Cells were extracted for 90 s at room temperature in a prewarmed (37 °C) extraction buffer containing 0.3% (v/v) Triton X-100 and 0.25% (wt/vol) glutaraldehyde in a microtubule-stabilizing buffer Kapitein (80 mM PIPES, 7 mM MgCl_2_, 1 mM egtazic acid, 150 mM NaCl, 5 mM d-glucose). The extraction buffer was replaced by a prewarmed (37 °C) fixation buffer (4% (wt/vol) paraformaldehyde in PBS) and incubated for 10 min at room temperature. The fixation buffer was removed by washing three times with PBS for 5 min under a small traveling wave in each chamber. After fixation, either the cell samples were stored in PBS with 50% (v/v) glycerol at 4 °C for up to 3 days or the samples were directly quenched.

Fluorescent quenching was performed by incubating 10 mM freshly prepared sodium borohydride in PBS for 7 min at room temperature. This was followed by a quick wash with PBS and two washes of 10 min on an orbital shaker. The fixed cells were permeabilized with 0.25% (v/v) Triton X-100 in PBS, incubated for 7 min at room temperature on the orbital shaker, and followed by three washes of 5 min on the orbital shaker with PBS. Fixed cells were blocked with a blocking buffer (BKK; 2% (wt/vol) bovine serum albumin, 10 mM glycine, 50 mM NH_4_Cl) either for 60 min at room temperature or overnight at 4 °C.

Primary and secondary antibodies or nanobodies were diluted in BKK according to the desired DOL. Incubation was done for each of the stainings for 1 h at room temperature in an incubation chamber and followed by three washes with BKK for 5 min on the orbital shaker. After the secondary staining and corresponding washes, the samples were postfixated by incubating for 10–15 min with 2% (wt/vol) paraformaldehyde in PBS. Finally, the fixated cells were washed thrice with PBS for 5 min on the orbital shaker. Samples were stored in 50% glycerol in PBS at 4 °C until used.

### Microscope Setup

Microscopic images were captured using a custom-built microscope based on the open microscope frame MiCube.^35^ Full details of the microscope setup can be found on Grußmayer Lab’s github page. In the excitation path, the setup incorporates a 1 W 638 nm laser (LAB-638–1000, Lasertack), which is delivered via a multimode optical fiber (NA 0.22, square core profile of size 70 μm by 70 μm, customized, Ceram Optec). The laser beam is then collimated by a 30 mm achromatic lens (AC254–030-A, Thorlabs) and focused by a 150 mm lens (147–643, Edmund Optics) onto the rear focal plane of an oil-immersion objective (NA 1.5, 60×, UPLAPO60XOHR, Olympus). A one-dimensional motorized stage (KMTS25E/M, Thorlabs) is incorporated to translate the collimated laser beam across the back focal plane, hence facilitating the transition among Epi, HILO, and TIRF illumination modalities. Furthermore, a vibration motor (5 mm Vibration Motor -11 mm Type, 304–111, precision microdrives) was used to agitate the optical fiber to ensure homogeneous laser intensity across the illumination area.^36^ Sample positioning is achieved via a three-dimensional Stick–Slip piezo stage (assembled by three identical linear stages, CLS5252, Smaract). Both the sample stage and the objective are fixed on the customized MiCube microscope body. Fluorescence is then decoupled from the excitation beam using a quad-band dichroic mirror (zt405/488/561/640rpc, Chroma) and further filtered by a notch filter (ZET405/488/561/640mv2, Chroma). A 180 mm tube lens (TTL80-A, Thorlabs) followed by two 300 mm lenses (G322336322, Qioptiq) in 4f configuration focused the image onto an sCMOS camera (BSI Express, Photometrics). A bandpass emission filter (ET706/95m, AHF Analysentechnik AG) was inserted for cleaning up the fluorescence of Atto 655. Images are acquired using μManager 2.0 gamma.

### Image Acquisition

Fixed cells were imaged in an imaging buffer containing 500 mM NaCl in PBS with varying imager strand concentrations at room temperature. In experiments exploring the impact of EC, 5% (v/v) EC (Fisher Scientific) was introduced into the imaging buffer.

Microtubule imaging was conducted by utilizing TIRF illumination, while HILO illumination was employed for imaging mitochondria. For each experiment, the selection of exposure time was based on a qualitative assessment of blinking kinetics and the SNR per frame, in combination with the resulting SOFI results. The specific imaging parameters for each image can be found in Supporting Information Table S1.

For the large FOV imaging, we used the multiposition acquisition in μManager where we generated 4 by 9 grids, with an overlap of 10% between tiles. 500 frames were recorded for one single tile before moving to the next grid. The grids were stitched together later reconstructing a large field-of-view image using the Stitching plugin on Fiji.^37^

### Data Analysis

#### SOFI Cross-Cumulant Analysis

The SOFI calculations were performed using a cross-cumulant-based algorithm available from https://www.github.com/kgrussmayer/sofipackage and implemented in MATLAB R2021b. Constant parameters were chosen to allow for comparison between the imaging buffer conditions. The input image sequence was subdivided into subsequences of 1000 frames each. This subsequent length was chosen to minimize the influence of photobleaching. If the input was fewer than 1000 frames, the subsequence length was set to match the total length of the imaging series. As a preprocessing step, drift correction based on cross-correlation between the different SOFI subsequences was applied. For postprocessing, deconvolution parameters were configured with a PSF approximation of a Gaussian with an fwhm of 4.2 pixels and a total of 10 iterations.

#### SMLM

The single-molecule localization microscopy (SMLM) reconstruction was conducted by using the ThunderSTORM plugin within FIJI. Default settings were applied for image filtering and the approximate localization of molecule parameters. Subpixel localization of molecules was achieved utilizing a PFS Integrated Gaussian approach, with the fitting radius set to 4 pixels and an initial sigma of 1.6 pixels, employing a weighted least squares fitting method.

Visualization of the reconstructed data was facilitated through the use of averaged shifted histograms magnified at 5.0× with an update frequency of 50 frames. Post localization, drift correction in the *xy* plane was performed using cross-correlation methods to ensure accurate spatial alignment. Additionally, single-molecule localizations with uncertainty values exceeding 15 were filtered out to enhance the data reliability and precision.

#### Decorrelation Analysis

The resolution of the SOFI results was evaluated based on image decorrelation analysis described by Descloux et al.^19^ This algorithm computes spatial resolution within a single image by employing partial phase autocorrelation, which involves the application of a mask filter and the computation of cross-correlation coefficients in Fourier space. To elaborate further, the analysis involves two primary steps. First, a normalized Fourier transform is calculated and subsequently cross-correlated with the original input image in Fourier space using a Pearson correlation. Second, this cross-correlation procedure is iteratively executed, while the Fourier transform is filtered through a binary circular mask featuring a diminishing radius ranging between 0 and 1.

In this work, the resolution of SOFI results was calculated using the MATLAB software from https://github.com/Ades91/ImDecorr. To ensure uniformity throughout the calculations, fixed settings were chosen, taking into account factors such as computational efficiency and precision. The normalized frequencies where the decorrelation curve has to be computed range from 0 to 1 with 100 equidistant points within this interval. The number of high-pass filters used to calculate the resolution was set to 20.

#### Microtubule Cross Sections

An alternative approach to assess the resolution is by evaluating the intensity profile of a cross section of converging microtubules. A perpendicular line profile was defined across a microtubule, and intensity values were recorded and normalized for each experimental condition (including the average intensity profile and higher-order SOFI images). This process necessitated appropriate scaling and considered pixel reduction resulting from SOFI postprocessing.

#### Jackknife SNR

SOFI-specific SNR characterization was performed to ensure sufficient image quality using Jackknife resampling.^18^ The algorithm was implemented as part of the SOFI cross-cumulative algorithm and is computationally expensive. Consequently, SNR estimation was conducted solely for specific, carefully chosen acquisitions.

Jackknife resampling involves creating *N* new data sets, where *N* corresponds to the number of raw images in the original data set. Each new data set is generated by excluding one image from the sequence and is subsequently employed to generate a new SOFI image, resulting in *N* new SOFI images. For every pixel value *I*(*x*, *y*) in the original SOFI image, *N* new values *I*_*n*_(*x*, *y*) are produced. These values provide a distribution of possible pixel intensities for that specific pixel location. The variation in these values across the new SOFI images provides insight into the uncertainty associated with the original pixel value. This uncertainty can then be used to calculate the SNR per pixel. The SNR per pixel is defined as
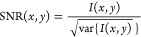
1Here, the uncertainty associated with the original pixel value is
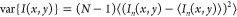
2

#### Intensity Time Traces

The methodology employed for intensity analysis involved the computation of normalized average pixel intensities over a temporal sequence. This analysis was conducted within a defined region of interest spanning 3 × 3 pixels across the entire time series. The selection of the specific pixel area involved the identification of a representative microtubule structure within the average wide-field projection image. This strategy aimed to ensure that the chosen region was relevant and reflective of the underlying sample characteristics.

#### Quantitative Analysis of Imaging Artifacts

SQUIRREL was used for the quantitative analysis of imaging artifacts for SOFI and SMLM reconstructions. Specifically calculating the resolution-scaled error (RSE) and the RSP correlation was done using the NanoJ (no GPU) Fiji plugin.^25^ The algorithm requires three inputs: a reference image (generally diffraction-limited), a super-resolution image, and a representative resolution scaling function (RSF) image. For the reference images, three separate wide-field images were generated for SOFI2, SOFI6, and SMLM, using the standard deviation of the frames included in each super-resolution reconstruction. To ensure pixel alignment, both the wide-field and SOFI images were cropped as required by the plugin. Following the method described by Culley et al., the plugin aligned the super-resolution images to the reference wide-field image and applied an RSF to match their resolutions.^25^ Finally, the RSP and the RSE between the two images were calculated for each input.

## Data Availability

The SOFI and SMLM data are openly available at https://github.com/klarinda/DNA-PAINT-SOFI.

## Supporting Information Available

Additional details on the theoretical background of SOFI, the buffer optimization process, and an overview of imaging parameters, including until sixth-order SOFI images of mitochondria, as well as sixth-order SOFI images using a nonspeed-optimized probe; resolution analysis of SOFI cumulants; evaluation of bleaching resistance; performance comparison of second- and fourth-order SOFI versus SMLM across different acquisition times; and a quantitative analysis of imaging artifacts (PDF)

## Notes

This work was previously submitted as a preprint: K.d.Z.; R.H.; M.N.F.H.; H.L.W.; M.T.; H.G.; K.G. High-Throughput Single Molecule Microscopy with Adaptable Spatial Resolution Using Exchangeable Oligonucleotide Labels. 2024, Article Number 2024.10.30.621026. bioRxiv. 10.1101/2024.10.30.621026 (accessed 02 12,2025).
