# Supplementary material for: High-Throughput Single-Molecule Microscopy with Adaptable Spatial Resolution Using Exchangeable Oligonucleotide Labels
Source: ACS Nano. 2025 Mar 27;19(13):13149–59. doi: 10.1021/acsnano.4c18502 (PMC11984304; doi:10.1021/acsnano.4c18502)
Supplement: Supplementary file 1 — nn4c18502_si_001.pdf [file nn4c18502_si_001.pdf]

Supporting Information: High-throughput single molecule microscopy with adaptable spatial resolution using exchangeable oligonucleotide labels

Klarinda de Zwaan[1], Ran Huo[1], Myron N.F. Hensgens[2], Hannah Lena Wienecke[1], Miyase Tekpınar[1], Hylkje Geertsema[2], and Kristin Grußmayer[1,*]

[1]Department of Bionanoscience, Kavli Institute of Nanoscience Delft, Delft University of Technology, 2629 HZ Delft, The Netherlands
[2]Department of Imaging Physics, Delft University of Technology, 2628 CJ Delft, The Netherlands
[*]k.s.grussmayer@tudelft.nl

Supporting Information

Supplementary note 1: SOFI theory

Super-resolution optical fluctuation imaging (SOFI) is a super-resolution technique that enhances spatial resolution by computing higher-order statistics of a time series of stochastically blinking fluorophores. SOFI does not demand major technical modifications to the microscope, e.g. a standard widefield microscope can be used, and is flexible regarding fluorophores and imaging conditions. The only requirement is that the sample has been labeled with fluorescent emitters that switch between two (or more) distinct states independently from each other and repeatedly in a stochastic way (1).

SOFI's super-resolving power stems from the assumption that fluorescent emitters switch independently and stochastically, correlating only in time and space with themselves. This independence, combined with the mathematical properties of cumulants (statistical quantities related to correlation functions), enables the extraction of higher-order statistical information from the blinking patterns of the fluorophores. The cumulants effectively filter out cross-terms and uncorrelated noise, allowing for the reconstruction of images with enhanced spatial resolution.

Fluorescent signal and cumulants

The detected intensity on the camera, assuming N single, independently fluctuating emitters, can be described by:

$$I(\mathbf{r},t) = \sum_{k=1}^{N} U(\mathbf{r}-\mathbf{r}_k) \cdot \varepsilon_k \cdot s_k(t) + b(\mathbf{r},t). \tag{1}$$

where $U(\mathbf{r})$ represents the optical systems point spread function (PSF), and $b(\mathbf{r},t)$ describes the background signal, including uncorrelated noise. The time-dependent molecular brightness is described for each emitter by $\varepsilon_k \cdot s_k(t)$, where ε_k denotes the constant molecular brightness and $s_k(t)$ represents a time-dependent fluctuation component with values of 0 (off-state) and 1 (on-state).

To capture detailed patterns of fluorescence signal fluctuations which contribute to the super-resolved image, we analyze nth-order cumulants. In general, spatio-temporal cumulants can be calculated using various time lags and different combinations of pixels to explore both temporal and spatial correlations in the fluorescence signal. We use a time lag of zero to simplify the analysis and cross-cumulants between different pixels to suppress shot noise (2, 3, 4). Our SOFI algorithm generates virtual pixels which are essentially interpolated points between the physical pixels captured by the camera. The higher-order cross-cumulants thus inherently provide a finer sampling of the super-resolved image. Mathematically, the nth-order cumulant with zero time lag can be written as:

$$\kappa_n\{I(\mathbf{r},t)\} = \sum_{k=1}^{N} U^n(\mathbf{r}-\mathbf{r}_k)\varepsilon_k^n \kappa_n\{s_k(t)\} + \kappa_n\{b(\mathbf{r},t)\}. \tag{2}$$

In the context of SOFI, the fluorophore's blinking behavior, $s_k(t)$, can be thought of as a binary function taking the value 1 when the fluorophore emits and 0 when it is off. Its cumulant, $\kappa_n\{s_k(t)\}$, is a Bernoulli distribution with probability ρ (i.e. on-time ratio) and can be approximated by an nth-order polynominal function of the on-time ratio $f_n(\rho) = (1-\rho)\frac{\partial f_n}{\partial \rho}$. Moreover, the term $\kappa_n\{b(\mathbf{r},t)\}$ for a constant background and additive noise is eliminated from higher-order cumulants because additive noise is assumed random and uncorrelated. Hence, we can estimate the nth-order cumulant with zero time lag as:

$$\kappa_n\{I(\mathbf{r},t)\} = \varepsilon_k \cdot f_n(\rho) \sum_{k=1}^{N} U^n(\mathbf{r}-\mathbf{r}_k) \cdot \varepsilon_k^n. \tag{3}$$

Resolution enhancement

The effective PSF in SOFI is raised to the power of n, leading to a narrowing of the PSF by a factor of $\sqrt{n}$, consequently resulting in enhanced spatial resolution. Using deconvolution techniques enables a final resolution improvement that scales linearly with the order of the cumulant. However, higher-order SOFI imaging comes with challenges, including the amplification of heterogeneities in molecular brightness ε^n, which may mask less bright structural details. To address this issue, Geissbuehler et al. proposed deconvolving the cumulant images and then linearizing the brightness response by applying the n-th root to the deconvolved cumulant image (2, 5).

It is important to note that while there are no theoretical limitations to calculating higher SOFI, the signal-to-noise ratio and signal-to-background ratio typically decrease, increasing the risk of processing artifacts. Additionally, computational complexity grows with the order n, leading to higher memory requirements and longer processing times.

Supplementary note 2: Buffer optimization

To accelerate both binding and unbinding rates in our experiments, we optimized the imaging buffer composition by increasing the imager strand concentration and incorporating ethylene carbonate (EC). This Supplementary Note details the buffer optimization process and its impact on the blinking kinetics.

Microtubules in fixed COS-7 cells were immunostained with a speed optimized docking strand (6). By increasing the imager strand concentration, the probability of hybridization of the DNA oligos is raised (Fig S1a,b). Compared to the initial imager strand concentration (Fig S1a), an increase in signal per frame is observed, indicating more frequent binding upon addition of imager strands. This is confirmed by evaluating the intensity time traces over time (Fig S2).

As for any fluorescence imaging, the SOFI signal contrast depends on the SNR and signal to background ratio (SBR), but also on the fluctuation statistics and sampling of the blinking. Solely increasing the imager strand concentration has limitations for high-order SOFI reconstructions, as it raises background noise, reducing the SNR and SBR. Moreover, when the off-time is reduced to the point of continuous occupation of resolvable binding sites, fewer intensity fluctuations are observed, consequently diminishing the SOFI signal. Cevoli et al. performed a parameter optimization for SOFI using a design of experiments approach, where simulated data was used to investigate the parameters influencing the quality of SOFI images (7). The analysis indicated that the best SOFI results were achieved when the fluorescent on- and off-times were minimized. Thus, to obtain more intensity fluctuations the on-time should also be minimized.

Prior research has demonstrated the acceleration of DNA dehybridization through the incorporation of ethylene carbonate (EC) (8). This aprotic solvent increases the solubility of the DNA bases and has been effectively employed to accelerate DNA-PAINT experiments by decreasing the on-time without affecting the off-time (9). Supplementing the high imager strand concentration buffer with EC leads again to the appearance of single blinks, signifying a notable reduction in on-time (Fig S1c, Fig S4). This observation is confirmed by comparing the intensity time traces over time: upon the addition of EC, single peaks are observed whereas without EC, the intensity fluctuations in the signal are less apparent (Fig S2). Further increasing the concentration of EC is eventually hindered due to changes in the refractive index of the buffer (data not shown).

Figure	Docking strand<br>Imager strand	Buffer composition:<br>[Imager strand], [EC]	Frames	Exposure time (s)	Illumination intensity (kW cm^{-2})	Resolution (nm)
Figure 2, S5, S6	5xR1<br>R1-Atto655	100 pM	10,000	30	2.5	74
Figure 3a, S7a SOFI 2	5xR1<br>R1-Atto655	20 nM, 5% EC	500	10	2.5	226
Figure 3b, S7b SOFI 4	5xR1<br>R1-Atto655	20 nM, 5% EC	5,000	10	2.5	169
Figure 3c, S7c SMLM	5xR1<br>R1-Atto655	50 pM	30,000	100	3.1	LP: 7<br>FWHM: 63
Figure 4b SOFI 2	5xR1<br>R1-Atto655	20 nM, 5% EC	500	10	1.2	230
Figure 4, S8 SOFI 2	5xR1<br>R1-Atto655	5 nM, 5% EC	1,000	30	3.1	221
Figure 4, S8 SOFI 6	5xR1<br>R1-Atto655	5 nM, 5% EC	8,000	30	3.1	74
Figure 4, S8 SMLM	5xR1<br>R1-Atto655	25 pM	15,000	100	3.1	LP: 8<br>FWHM: 65
Figure S1a, S2a	5xR1<br>R1-Atto655	100 pM	10,000	50	2.5	NA
Figure S1b, S2b	5xR1<br>R1-Atto655	5 nM	10,000	10	2.5	NA
Figure S1c, S2c	5xR1<br>R1-Atto655	5 nM, 5% EC	10,000	30	3.1	NA
Figure S3	5xR1<br>R1-Atto655	5 nM, 5% EC	10,000	50	3.1	73
Figure S4	P3<br>P3-Atto655	10 nM, 5% EC	10,000	30	3.1	115

Table S1. Overview of sample types, buffer conditions, imaging parameters, and achieved resolutions. For SOFI images, resolution is assessed through decorrelation analysis, with the resolution of 6th order SOFI presented unless stated otherwise in the associated figure. For SMLM reconstructions, localization precision (LP) and full width half maximum (FWHM) are provided.

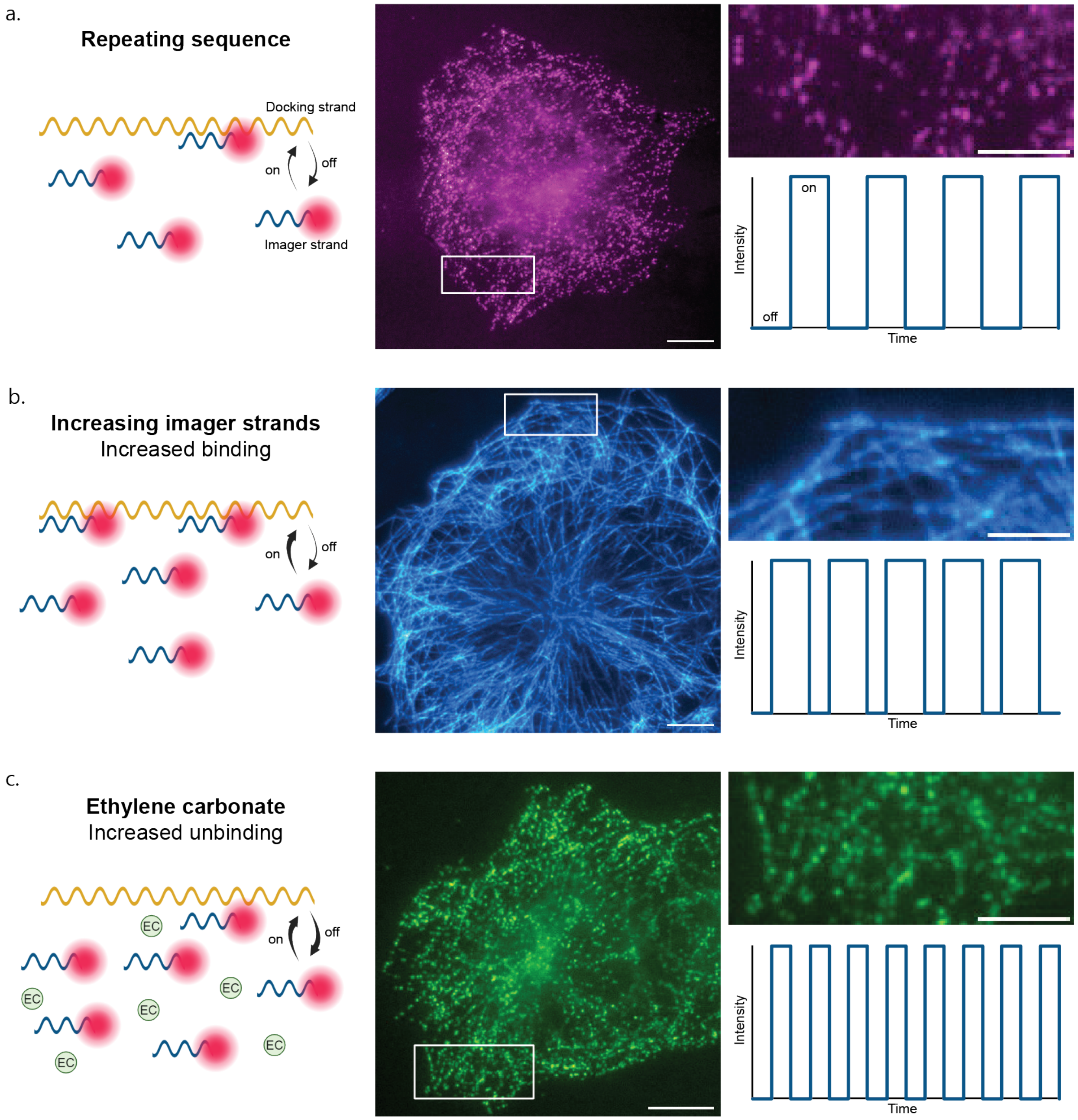

Figure S1. Influence of buffer composition on blinking kinetics. (a) Raw image frame displaying blinking behavior under low concentration of imager strands of COS7 cells stained for microtubules with repeating docking sequence (6) (scale bar 10 μm). Close up of indicated area (scale bar 5 μm) and representation of corresponding blinking intensity time trace (see Fig S2) (b) Blinking behavior with increased imager strand concentration yielding an increased binding frequency. (c) Blinking behavior of increased imager strand concentration and addition of ethylene carbonate yielding both an increased binding and unbinding frequency.

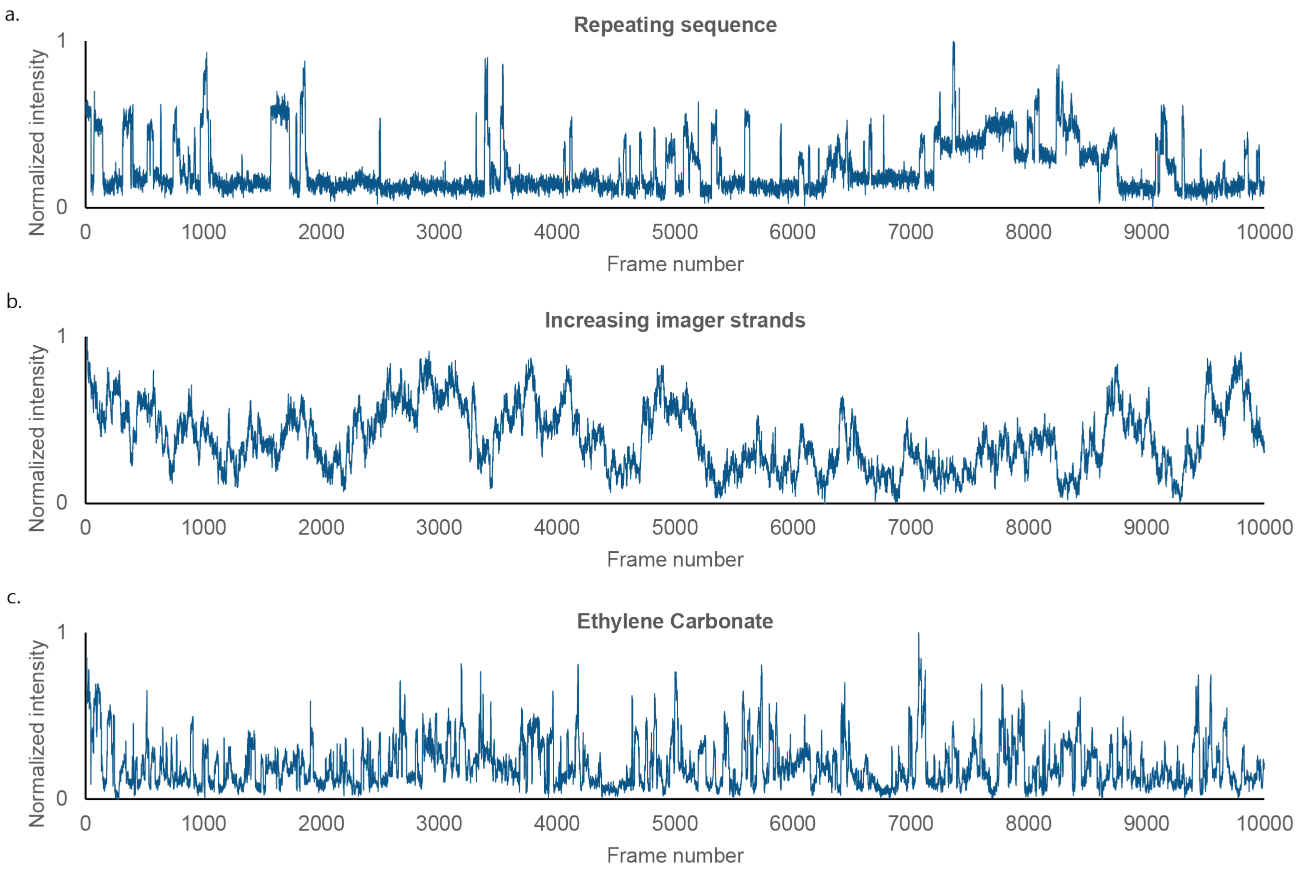

Figure S2. Intensity time traces for varying buffer conditions Intensity time traces of the signal at a microtubule for varying buffer compositions. (a) 100 pM imager strand (b) 5 nM imager strand (c) 5nM imager strand + 5% ethylene carbonate. Conditions correspond to the examples shown in Supplementary Fig S1. See Supplementary Table S1 for details about the imaging parameters.

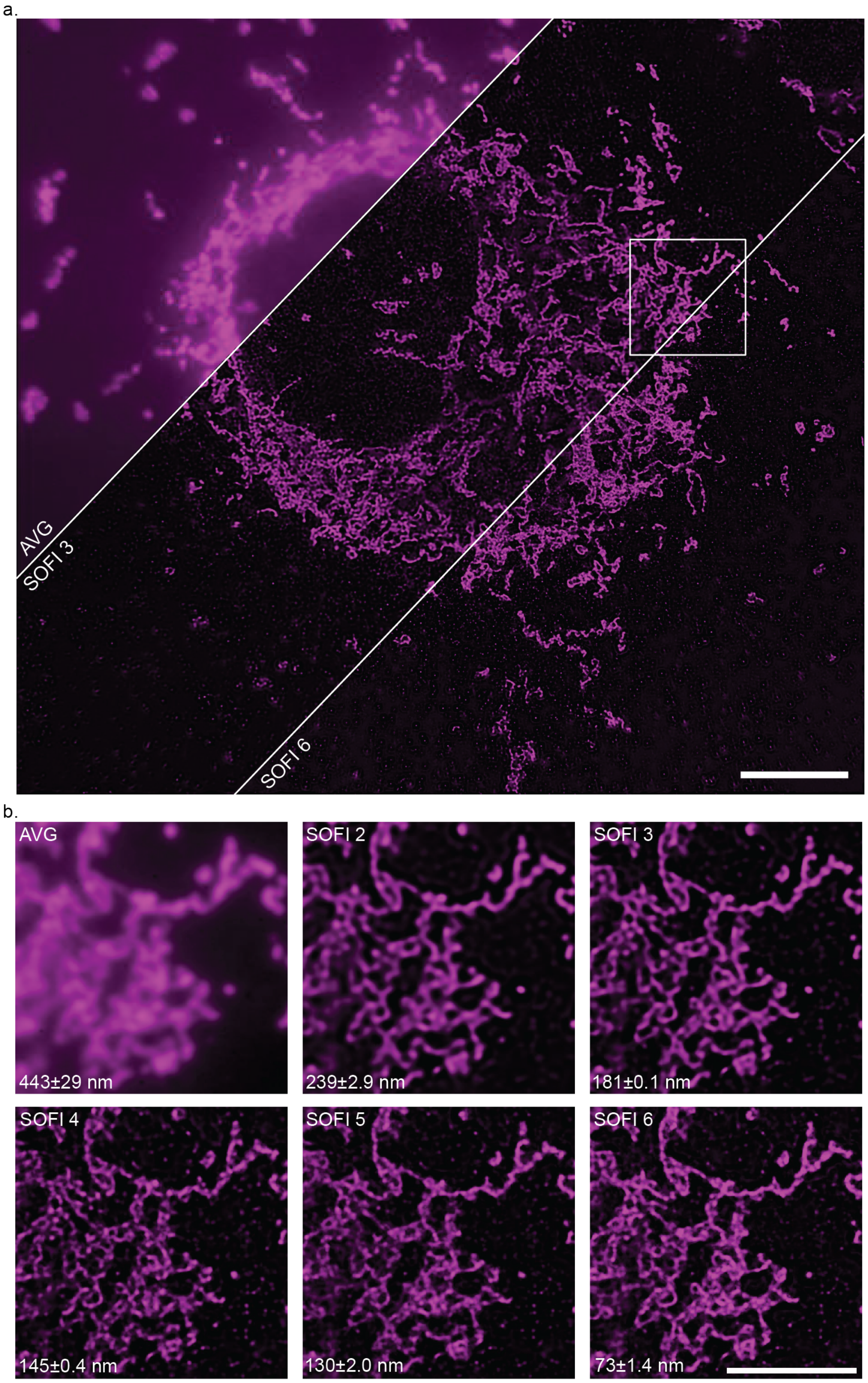

Figure S3. Up till 6th order SOFI imaging of mitochondria COS7 cells stained for mitochondria with repeating docking sequence and addition of ethylene carbonate. (a) Widefield (average of the image sequence), 3rd and 6th order SOFI as indicated (scale bar 10 μm) (b) Close-ups of diffraction limited average projection of the image sequence, 2nd, 3rd, 4th, 5th and 6th order SOFI as indicated (scale bar 5 μm). See Supplementary Table S1 for details about the imaging parameters.

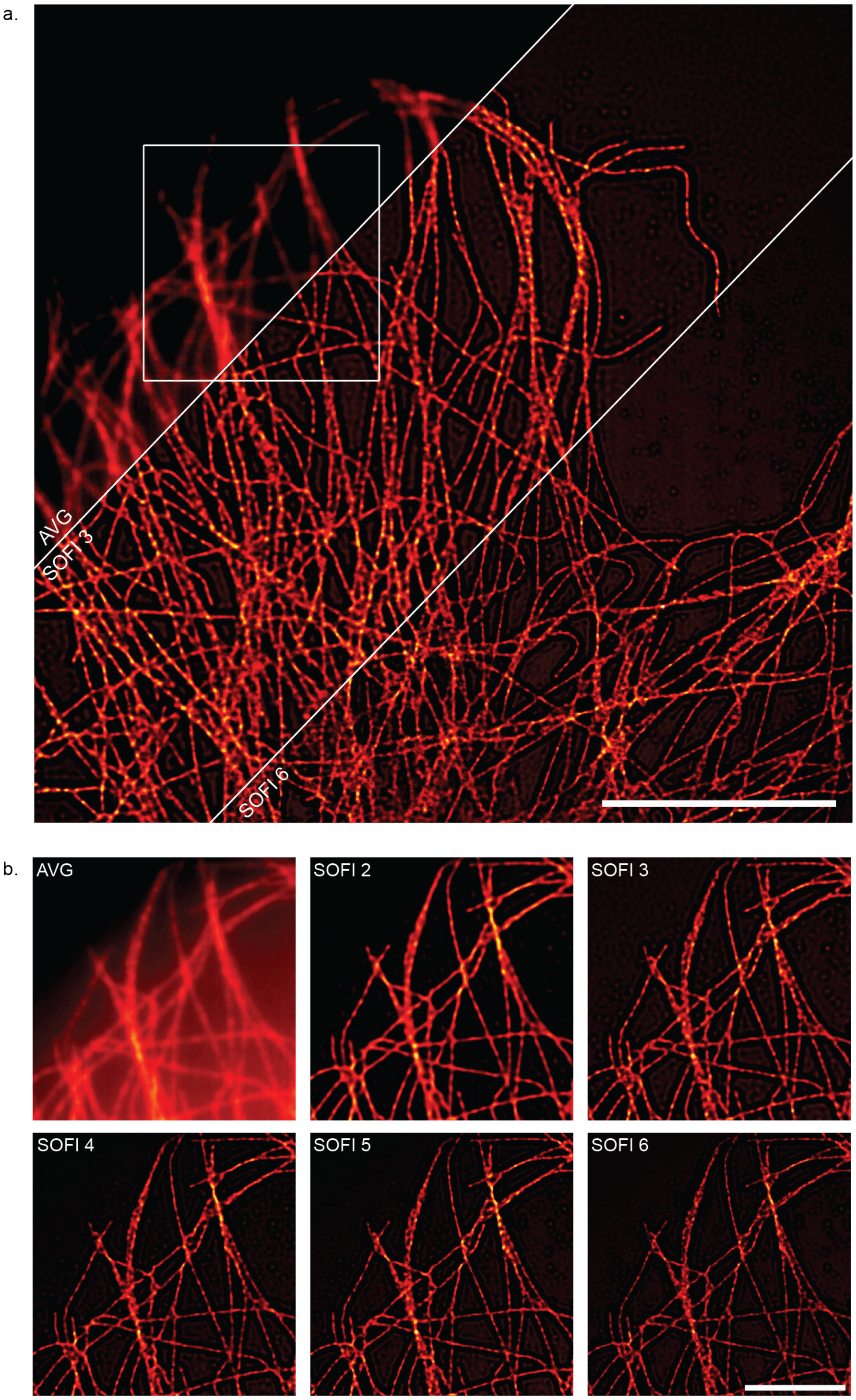

Figure S4. Up till 6th order SOFI imaging using non-speed optimized docking sequence COS7 cells stained for mitochondria with non-speed optimized docking sequence (P3) and addition of ethylene carbonate. (a) Widefield (average of the image sequence), 3rd and 6th order SOFI as indicated (scale bar 10 μm) (b) Close-ups of diffraction limited average projection of the image sequence, 2nd, 3rd, 4th, 5th and 6th order SOFI as indicated (scale bar 5 μm). See Supplementary Table S1 for details about the imaging parameters.

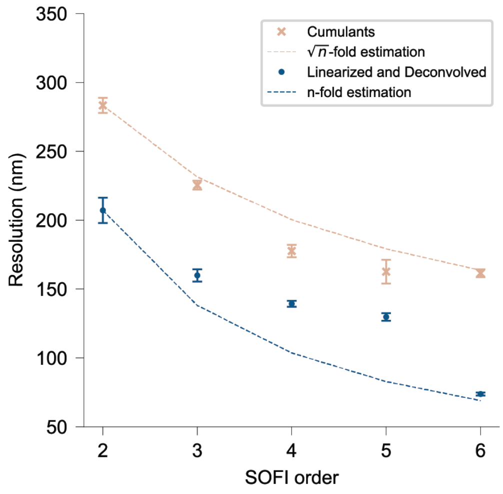

Figure S5. Resolution estimates for three different cells Resolution estimate using decorrelation analysis of SOFI cumulants (pink, average ± standard deviation) and subsequently linearized and deconvolved SOFI images (blue, average ± standard deviation). The dashed lines connect the theoretical resolution enhancements starting from the 2nd order SOFI image (pink $\sqrt{n}$- and blue n-fold improvement). See Supplementary Table S1 for details about the imaging parameters.

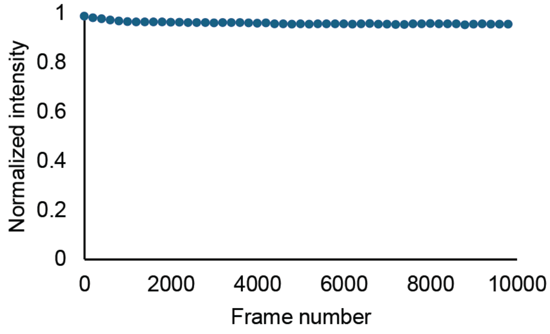

Figure S6. Bleaching resistance of DNA-PAINT for SOFI Mean fluorescence signal of full time series of three different cells stained for microtubules with DNA-PAINT. See Supplementary Table S1 for details about the imaging parameters.

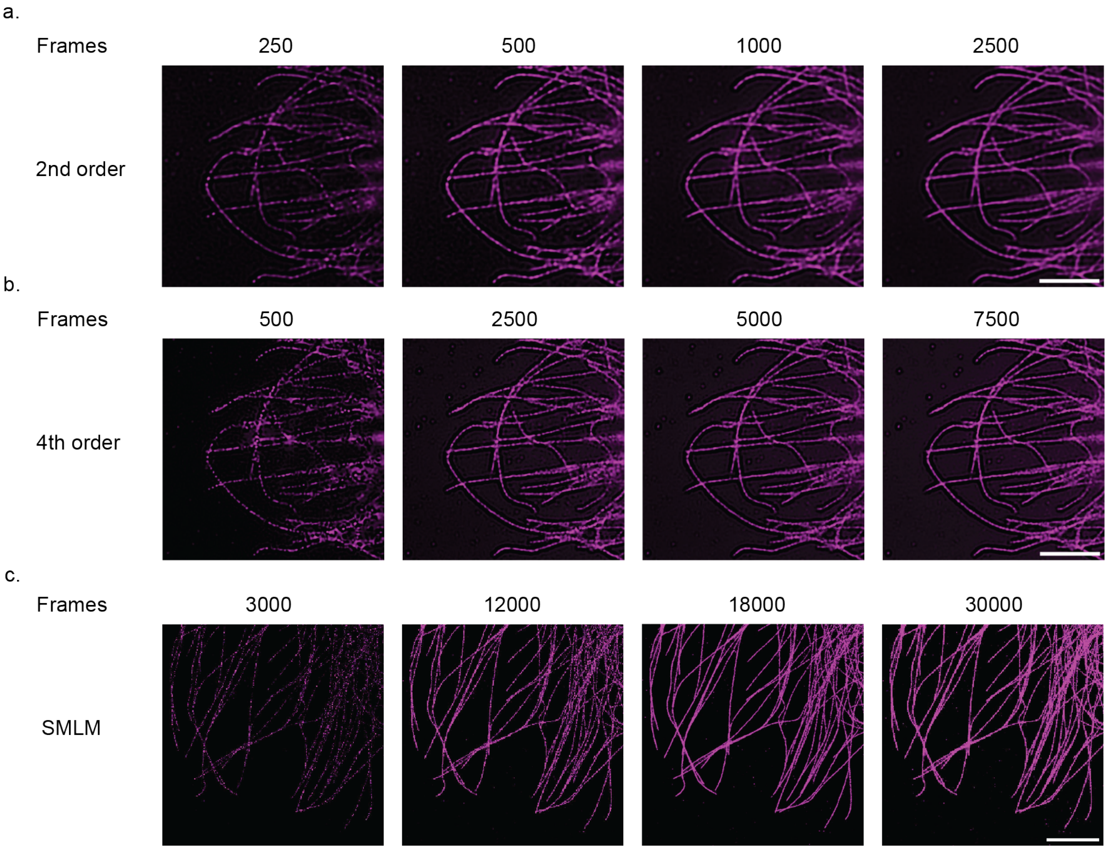

Figure S7. Performance comparison of 2nd and 4th order SOFI and SMLM with increasing acquisition time (a) 2nd order, (b) 4th order SOFI reconstructions and (c) SMLM reconstructions using the specified number of frames as input. See Supplementary Table S1 for details about the imaging parameters.

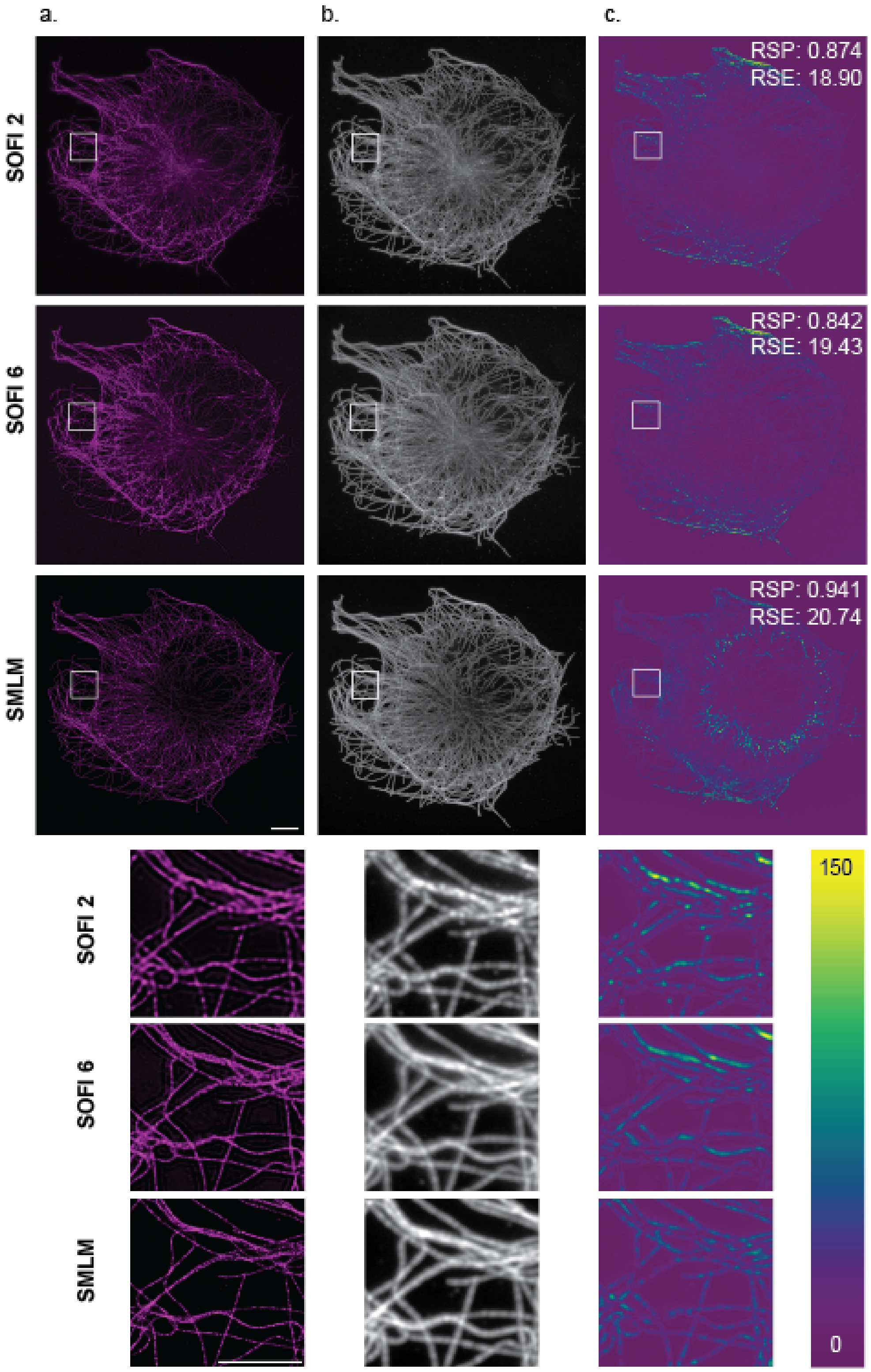

Figure S8. **Quantitative analysis of imaging artifacts** (a) Super resolution reconstruction for 2nd, 6th order SOFI and SMLM reconstruction as in Figure 4 (scale bar 10μm). Close up of indicated region for each reconstruction (scale bar 5μm). (b) Corresponding standard deviation widefield image of the frames included in each super resolution reconstruction (c) Quantitative map of errors between the standard deviation widefield image and convolved super resolution images using the SQUIRREL algorithm (10). Resolution Scaled Error (RSE) and Resolution Scaled Pearson coefficient (RSP) are denoted. The image processing of SMLM and SOFI among others provides some degree of optical sectioning. This violates the assumption in SQUIRREL that both reference and super-resolution images represent the same focal plane and thus accounts for part of the detected errors. This has been studied in detail for SMLM and SRRF (10, 11). See Supplementary Table S1 for details about the imaging parameters.

References

1. Dertinger, T., Colyer, R., Iyer, G., Weiss, S. & Enderlein, J. Fast, background-free, 3d super-resolution optical fluctuation imaging (sofi) (2009).

2. Geissbuehler, S. *et al.* Mapping molecular statistics with balanced super-resolution optical fluctuation imaging (bsofi). *Opt. Nanoscopy* **1**, 1–7, DOI: 10.1186/2192-2853-1-4 (2012).

3. Girsault, A. *et al.* Sofi simulation tool: A software package for simulating and testing super-resolution optical fluctuation imaging. *PLOS ONE* **11**, e0161602, DOI: 10.1371/JOURNAL.PONE.0161602 (2016).

4. Dertinger, T., Weiss, S., Enderlein, J., Vogel, R. & Colyer, R. Achieving increased resolution and more pixels with superresolution optical fluctuation imaging (sofi). *Opt. Express, Vol. 18, Issue 18, pp. 18875-18885* **18**, 18875–18885, DOI: 10.1364/OE.18.018875 (2010).

5. Geissbuehler, S. *et al.* Live-cell multiplane three-dimensional super-resolution optical fluctuation imaging. *Nat. Commun. 2014 5:1* **5**, 1–7, DOI: 10.1038/ncomms6830 (2014).

6. Strauss, S. & Jungmann, R. Up to 100-fold speed-up and multiplexing in optimized dna-paint. *Nat. Methods 2020 17:8* **17**, 789–791, DOI: 10.1038/s41592-020-0869-x (2020).

7. Cevoli, D. *et al.* Design of experiments for the optimization of sofi super-resolution microscopy imaging. *Biomed. Opt. Express* **12**, 2617–2630, DOI: 10.1364/BOE.421168 (2021).

8. Andrews, R. Dna hybridisation kinetics using single-molecule fluorescence imaging. *Essays Biochem.* **65**, 27, DOI: 10.1042/EBC20200040 (2021).

9. Civitci, F. *et al.* Fast and multiplexed superresolution imaging with dna-paint-ers. *Nat. Commun. 2020 11:1* **11**, 1–8, DOI: 10.1038/s41467-020-18181-6 (2020).

10. Culley, S. *et al.* Quantitative mapping and minimization of super-resolution optical imaging artifacts. *Nat. Methods 2018 15:4* **15**, 263–266, DOI: 10.1038/nmeth.4605 (2018).

11. Laine, R. F. *et al.* High-fidelity 3d live-cell nanoscopy through data-driven enhanced super-resolution radial fluctuation. *Nat. Methods 2023 20:12* **20**, 1949–1956, DOI: 10.1038/s41592-023-02057-w (2023).
